# *Bacillus aryabhattai* and silicon modulate soil biological activity and performance of *Mimosa caesalpiniifolia* under water deficit

**DOI:** 10.1007/s42770-026-01912-0

**Published:** 2026-03-16

**Authors:** Kele da Silva Mota, Kaio Gráculo Vieira Garcia, Murilo de Sousa Almeida, Francisco Luan Almeida Barbosa, Wardsson Lustrino Borges, Rosilene Oliveira Mesquita, Ademir Sergio Ferreira Araujo, Geocleber Gomes de Sousa, Arthur Prudêncio de Araujo Pereira

**Affiliations:** 1 Department of Soil Science, Federal University of Ceará, Ave. Mister Hull, 2977, Fortaleza, Ceará 60.021-970 Brazil; 2 Embrapa Tropical Agroindustry, Brazilian Agricultural Research Corporation, Fortaleza, Ceará Brazil; 3 Department of Plant Science, Federal University of Ceará, Fortaleza, Ceará Brazil; 4 Federal University of Piauí, Campus Ministro Petrônio Portella, Teresina, Piauí Brazil; 5 University for International Integration of the Afro-Brazilian Lusophony, Redenção, Ceará Brazil

**Keywords:** Plant-microbe interaction, Semiarid ecosystem, Water use efficiency, Soil respiration, Beneficial rhizobacteria

## Abstract

*Mimosa caesalpiniifolia* is a native legume commonly used in agroforestry systems, degraded land recovery, and timber production in semiarid regions. Although this species is naturally hardy, its seedlings are highly sensitive to water deficit during early growth stages, limiting their establishment under drought conditions. This study investigated the effects of *Bacillus aryabhattai* inoculation and silicon (Si) application on seedling growth, physiological performance, and soil microbial and enzymatic activity under two water regimes (50% and 100% water level) and four treatments (control, *B. aryabhattai*, Si, and *B. aryabhattai* + Si), in a greenhouse experiment. Seedlings under 100% water level showed significantly higher shoot and root biomass, leaf number, root length, plant height, and seedling quality indices. Chlorophyll and carotenoid contents increased in combined *B. aryabhattai* + Si treatments, independent of water level. Water use efficiency improved under drought conditions (50%). Soil basal respiration rose by ~ 87% and 75% with *B. aryabhattai* and *B. aryabhattai* + Si at 50% water level, respectively. Enzymatic activities (arylsulfatase, β-glucosidase, phosphatases, urease) increased up to 600% with *B. aryabhattai* + Si compared to control. Si concentration was highest in shoots with *B. aryabhattai* at 100% water level, and in roots with *B. aryabhattai* + Si at 50% water level. Soil Si concentration peaked in the Si-only treatment at 100% water level. These findings demonstrate the synergistic potential of *B. aryabhattai* and Si to enhance drought tolerance and soil biological quality in *M. caesalpiniifolia* seedlings, supporting sustainable restoration in semiarid ecosystems.

## Introduction

*Mimosa caesalpiniifolia*, commonly known as Sabiá, is a native Brazilian leguminous tree with multiple uses, including the restoration of degraded areas, agroforestry systems, timber production, landscaping, and forage. Despite its ecological potential and adaptability, the species is particularly sensitive to water scarcity during its early growth stages, which may compromise seedling survival and establishment in the field.

Nowadays, water scarcity is a major constraint on productivity in reforestation and agroforestry systems across the globe, especially during the early stages of plant establishment. Even slight decreases in soil moisture can lead to significant reductions in seedling growth, with losses reported up to 7.7% [1]. Importantly, plant responses to drought are highly context-dependent, varying according to environmental conditions, soil properties, and species-specific functional traits. This problem has been intensified by climate change, which promotes increasing rainfall irregularity and more frequent drought events, posing significant challenges to agricultural development, especially in tropical and semiarid regions. In these environments, rainfall variability directly affects the establishment of crops and forest species. Particularly to semiarid regions, water deficit severely impairs biomass accumulation and survival rates of young plants [2]. In these environments, water deficit directly affects the establishment of perennial species, disrupting physiological and morpho-anatomical processes, and often leading to reduced biomass production, impaired nutrient uptake, and limited root growth [3].

In this context, strategies that enhance plant resilience to water deficit have gained increasing attention. Among them, the use of plant growth-promoting microorganisms (PGPM), including beneficial rhizobacteria such as *Bacillus aryabhattai*, has emerged as a promising approach [4]. Showed that strains of *Bacillus* can induce drought tolerance through phytohormone production, enhanced nutrient uptake, and activation of antioxidant defense mechanisms. Moreover, these microorganisms can modify the rhizosphere, contributing to a balanced soil microbial community and improved plant performance under adverse conditions [5]. However, many of these findings derive from studies conducted under controlled or temperate conditions [6, 7], and their effectiveness under semiarid environments remains variable due to high temperatures, strong soil moisture fluctuations, and nutrient-poor soils [8, 9]. This environmental dependency highlights the need for context-specific evaluations [10].

Complementary to microbial strategies, silicon (Si) has been widely recognized for its beneficial effects on plant resistance to water deficit. It contributes to the formation of physical barriers that reduce water loss, strengthens plant tissues, and activates antioxidant systems that help mitigate drought-induced damage [11, 12]. Nevertheless, the magnitude of Si benefits differs among species and environmental conditions, and positive responses reported in agricultural crops or controlled environments are not always replicated under semiarid field conditions [13–15]. Interestingly, recent studies have highlighted the synergistic effects of Si and soil microorganisms, promoting improvements in plant growth, soil health, and tolerance to abiotic stress [16, 17]. Thus, the combination of Si and *B. aryabhattai* may represent an integrated strategy to support the development of vigorous seedlings even under limited water availability.

Despite these advances, little is known about the interactive effects of *B. aryabhattai* and Si on native forest species such as *M. caesalpiniifolia*, particularly under varying water availability. Most previous studies have focused on agricultural crops or single-factor approaches, limiting the ecological relevance of their findings for native forest restoration [18–20]. Moreover, how these strategies influence rhizosphere microbial activity under drought conditions is still poorly understood. This limitation is especially relevant in semiarid regions, where restoration outcomes rely on the establishment of drought-tolerant seedlings and the preservation of soil biological functions [21]. Understanding these interactions is crucial for developing integrated, sustainable approaches to seedling production in water-limited environments. Therefore, investigating the combined effects of PGPM and Si under water deficit is not only scientifically relevant but also ecologically meaningful for semiarid restoration programs.

Within this framework, the present study sought to assess the impacts of *B. aryabhattai* inoculation and Si application on the growth, physiological performance, and soil biological activity of *M. caesalpiniifolia* seedlings under different levels of water stress. We hypothesized that the combination of *B. aryabhattai* and Si would synergistically promote plant growth and physiological performance, by enhancing drought tolerance and stimulating biological activity in the soil, ultimately leading to more vigorous *M. caesalpiniifolia* seedlings under water-limited conditions.

## Materials and methods

### Experimental area and soil characterization

The study was conducted in a greenhouse facility within the Department of Soil Science, using soil collected from an area belonging to the Teaching and Research Center for Urban Agriculture, both located at the Federal University of Ceará (UFC), Pici Campus, Fortaleza, Brazil. During the study, average conditions inside the greenhouse were approximately 29 °C for air temperature and 75% for relative humidity. The polyethylene greenhouse cover used in our experiment transmitted ~ 70–80% of incident photosynthetically active radiation (PAR). Soil sampling was performed at a depth of 0.0–0.2 m. Following collection, chemical and physical analyses were carried out by the laboratory of the Ceará Foundation for Meteorology and Water Resources (FUNCEME).

The soil used in the experiment had the following chemical and physical characteristics: pH in water of 4.5; K⁺ content of 0.18 cmolc kg⁻¹, Ca²⁺ of 2.40 cmolc kg⁻¹, Mg²⁺ of 0.40 cmolc kg⁻¹, Na⁺ of 0.06 cmolc kg⁻¹, H⁺ + Al³⁺ of 4.46 cmolc kg⁻¹, and Al³⁺ of 0.50 cmolc kg⁻¹. The sum of bases (SB) was 3.04 cmolc kg⁻¹, the total cation exchange capacity (CEC) was 7.44 cmolc kg⁻¹, and the base saturation (V%) was 41.0%. The organic matter (OM) content was 21.39 g kg⁻¹, with 12.49 g kg⁻¹ of carbon (C), 1.36 g kg⁻¹ of nitrogen (N), and a C/N ratio of 9. Regarding particle size distribution, the soil contained 37.0 g kg⁻¹ of clay, 868.0 g kg⁻¹ of sand, and 95.0 g kg⁻¹ of silt. In summary, soil pH was measured using a 1:2.5 soil-to-water ratio. Exchangeable calcium (Ca²⁺), magnesium (Mg²⁺), and aluminum (Al³⁺) were extracted with 1 mol L⁻¹ KCl solution, while potential acidity (H + Al) was determined using 0.5 mol L⁻¹ calcium acetate buffered at pH 7. The cation exchange capacity (CEC) was estimated by summing Ca²⁺, Mg²⁺, K⁺, H⁺, and Al³⁺ concentrations, and the sum of bases (SB) was calculated from the combined values of Ca²⁺, Mg²⁺, K⁺, and Na⁺. Base saturation (V%) was derived as the ratio of SB to CEC, expressed as a percentage. Soil texture (clay, sand, and silt contents) was determined by the pipette method following the methodology described by [22].

### Experimental design

A completely randomized design was used in a two-factor factorial arrangement, consisting of: (i) two irrigation levels (50% and 100% of field capacity), and (ii) four treatments: control (no inoculant or Si), *B. aryabhattai*, Si, and the combined application of *B. aryabhattai* + Si. Each treatment was replicated four times, totaling 32 experimental units.

### Definition of water levels based on soil water parameters

To establish the water levels of 50% and 100%, the field capacity (FC) and permanent wilting point (PWP) of the soil were determined in triplicate at the Soil Physics Laboratory of UFC. FC was measured using a tension table at 10 kPa, and PWP using a Richards chamber at 1500 kPa [23].

Based on these values, the amount of water needed for irrigation was determined based on the following equation:

$$\mathrm{VI}={\left(\mathrm{Vp}-\mathrm{Vd}\right)}/{\left(1-\mathrm{L}\right)}$$

Where VI is the volume of water to be applied (mL); Vp is the volume previously applied (mL); Vd is the volume of drained water (mL); and L is the leaching fraction (0.15).

Water levels were adjusted based on the drainage lysimeter principle [24], maintaining the soil moisture at 100% (high water level) for the control and at 50% (low water level) for the water stress treatment, with continuous monitoring of soil moisture throughout the experimental period.

### Experimental setup

The cultivation of *Mimosa caesalpiniifolia* was carried out in pots containing 3 kg of soil previously characterized for its chemical properties. Based on these data, soil acidity was corrected using dolomitic lime (with 95% relative neutralizing power), aiming to achieve a base saturation (V%) of 50%. After a 15-day incubation period, Si was applied. For this purpose, 200 mg kg⁻¹ of Si was incorporated into the soil using sodium silicate (Na₂SiO₃) as the source, with the amount determined based on previous studies [25, 26]. After application, the soil was thoroughly mixed and incubated for 7 days.

Before sowing, *M. caesalpiniifolia* seeds were immersed in 70% ethanol for 30 s to reduce surface tension. Afterwards, the seeds were disinfected in a 1% sodium hypochlorite solution for 10 min and then rinsed multiple times with sterile distilled water to remove any residual hypochlorite [27, 28]. After surface disinfection, seeds were inoculated with the bacterial strain *Bacillus aryabhattai* CMAA 1363, a component of the commercial bioinput AURAS^®^, developed by Embrapa in collaboration with NOOA Ciência e Tecnologia. The product was applied at a rate of 4 mL per kg of *M. caesalpiniifolia* seeds and homogenized to ensure uniform distribution of the inoculant. AURAS^®^ contains the microorganism at a density of 1 × 10⁸ CFU mL⁻¹.

Four seeds were planted in each pot, and one week after germination, seedlings were thinned to leave a single plant per experimental unit. *M. caesalpiniifolia* seedlings were grown in a greenhouse for up to 90 days after emergence (DAE).

### Analytical measurements

#### Growth parameters

After 90 days DAE, plants were collected and separated into leaves, stems, and roots, then placed in paper bags for further analysis. At harvest, growth variables recorded included height, leaves count, stem diameter, root length, and dry biomass. Shoot height was measured from the soil surface to the tip of the main stem’s terminal bud using a ruler (cm). Stem diameter was taken 5 cm above the root collar with a digital caliper calibrated to millimeter accuracy. Root length was also measured with a ruler in centimeters. For determination of dry biomass, the shoot and root samples were dried in a forced-air oven at 60 °C until constant weight was achieved. The dry mass of the shoot (SDM) and root (RDM) was then recorded using an analytical balance.

#### Seedling quality

Seedling vigor was assessed using the Dickson Quality Index [29] and the Robustness Index [30], both calculated based on the collected morphological data. The following equations were applied:

1 $$\mathrm{D}\mathrm{i}\mathrm{c}\mathrm{k}\mathrm{s}\mathrm{o}\mathrm{n}\;\mathrm{Q}\mathrm{u}\mathrm{a}\mathrm{l}\mathrm{i}\mathrm{t}\mathrm{y}\;\mathrm{I}\mathrm{n}\mathrm{d}\mathrm{e}\mathrm{x}=\frac{\mathrm{T}\mathrm{o}\mathrm{t}\mathrm{a}\mathrm{l}\;\mathrm{D}\mathrm{r}\mathrm{y}\;\mathrm{M}\mathrm{a}\mathrm{s}\mathrm{s}}{\left(\frac{\mathrm{S}\mathrm{t}\mathrm{e}\mathrm{m}\;\mathrm{D}\mathrm{i}\mathrm{a}\mathrm{m}\mathrm{e}\mathrm{t}\mathrm{e}\mathrm{r}}{\mathrm{H}\mathrm{e}\mathrm{i}\mathrm{g}\mathrm{h}\mathrm{t}}\right)+\left(\frac{\mathrm{S}\mathrm{h}\mathrm{o}\mathrm{o}\mathrm{t}\;\mathrm{D}\mathrm{r}\mathrm{y}\;\mathrm{M}\mathrm{a}\mathrm{s}\mathrm{s}}{\mathrm{R}\mathrm{o}\mathrm{o}\mathrm{t}\;\mathrm{D}\mathrm{r}\mathrm{y}\;\mathrm{M}\mathrm{a}\mathrm{s}\mathrm{s}}\right)}$$

2 $$\mathrm{R}\mathrm{o}\mathrm{b}\mathrm{u}\mathrm{s}\mathrm{t}\mathrm{n}\mathrm{e}\mathrm{s}\mathrm{s}\;\mathrm{I}\mathrm{n}\mathrm{d}\mathrm{e}\mathrm{x}=\frac{\mathrm{H}\mathrm{e}\mathrm{i}\mathrm{g}\mathrm{h}\mathrm{t}}{\mathrm{S}\mathrm{t}\mathrm{e}\mathrm{m}\;\mathrm{D}\mathrm{i}\mathrm{a}\mathrm{m}\mathrm{e}\mathrm{t}\mathrm{e}\mathrm{r}}$$

#### Physiological parameters

Water use efficiency (WUE) was measured at 90 DAE, between 09:00 and 11:00 a.m., using fully expanded leaves. Measurements were taken using an infrared gas analyzer (Li-6400XT; Li-Cor, Lincoln, NE, USA) operating in an open system with an airflow rate of 300 mL min⁻¹. WUE was expressed as the ratio of net CO₂ assimilation to transpired water (µmol CO₂ mmol⁻¹ H₂O), indicating the plant’s water-use capacity.

Quantification of chlorophyll a, chlorophyll b, total chlorophyll, and carotenoids was performed in accordance with the protocol described by [31]. Three leaf discs (1.0 cm in diameter) were collected from the first fully expanded leaf pair and placed in test tubes containing 5 mL of dimethyl sulfoxide (DMSO) saturated with CaCO₃. The tubes were wrapped in aluminum foil, sealed, and incubated in a water bath at 65 °C for 30 min. After cooling to room temperature, the pigment extracts were used for spectrophotometric readings at specific wavelengths.

#### Microbial activity

Soil basal respiration (SBR) was assessed using the method adapted from [32]. To accomplish this, 100 g of moist soil was placed in sealed incubation flasks together with 20 mL of 0.5 N NaOH to capture the CO₂ emitted from microbial activity. CO₂ measurements were taken daily over a 7-day period, with the NaOH solution being replaced every 24 h. To quantify microbial biomass carbon (MBC), the study employed the fumigation-extraction technique described in [33]. Soil samples (20 g) were fumigated with ethanol-free chloroform for 24 h. MBC values were estimated considering the C difference between fumigated and unfumigated samples, applying a correction factor equal to 0.33. The metabolic quotient (*q*CO₂) was calculated as the ratio of SBR to MBC.

#### Soil enzyme activity

Enzymatic activities in the soil were assessed using colorimetric methods that detect the release of either *p*-nitrophenol (PNP) or ammonium ions (NH₄⁺), depending on the specific substrate-enzyme interaction. β-Glucosidase activity was measured by incubating soil samples with a buffered solution of p-nitrophenyl-β-D-glucoside. The amount of *p*-nitrophenol released during the reaction served as an indicator of enzyme activity [34]. Arylsulfatase activity was determined by incubating soil with p-nitrophenyl sulfate potassium salt in an acetate buffer adjusted to pH 5.8 [35]. A volume of 4 mL of buffer was added to each sample, and enzyme activity was quantified based on the colorimetric detection of *p*-nitrophenol released during the reaction. Phosphatase activity, both acid and alkaline, was assessed using *p*-nitrophenyl phosphate (PNP) as a substrate. Reactions were performed in 4 mL of modified universal buffer (MUB), with the pH adjusted to 6.5 for acid phosphatase and to 11.0 for alkaline phosphatase. The amount of *p*-nitrophenol produced was used to quantify enzyme activity [34]. Urease activity was measured by quantifying the ammonium ions released following the incubation of soil samples with a urea solution at pH 10. In these alkaline conditions, urea undergoes hydrolysis to produce ammonia (NH₃), which then reacts with water to form ammonium hydroxide (NH₄OH), subsequently dissociating into NH₄⁺ and OH⁻ ions. The released NH₄⁺ was measured colorimetrically as an indicator of urease activity [36].

#### Si content in shoots, roots, and soil

Si concentration in shoot and root tissues was determined following the method of [37]. Dried and ground samples (0.1 g) of shoots or roots were digested with 2 mL of 30% H₂O₂ and 3 mL of 50% NaOH in Falcon tubes. The mixture was heated in a water bath at 85 °C for 1 h, followed by autoclaving at 123 °C and 1.5 atm for an additional hour. After digestion, samples were filtered and subsequently analyzed using a spectrophotometer at a wavelength of 410 nm. Available Si in the soil was determined following the method described by [38]. Dried and sieved soil samples (10 g) were transferred to plastic Erlenmeyer flasks, and 100 mL of 0.5 mol L⁻¹ acetic acid solution were added. The mixture was shaken for 1 h on a horizontal shaker. Afterwards, the samples were filtered, and the Si concentration was quantified by spectrophotometry at a wavelength of 660 nm.

### Statistical procedures

The entire dataset underwent preliminary evaluation for statistical assumptions, with data normality distribution assessed through the Shapiro-Wilk procedure and homogeneity of variance checked by Levene’s test. Following these assessments, analysis of variance (ANOVA) was conducted via the F-test (*p* < 0.05). When significant differences were detected, mean comparisons were performed using the Scott-Knott test at the same confidence level. The statistical analysis was performed using AgroEstat software (version 1.1.0.712) [39]. Spearman’s rank correlation analysis was performed to assess relationships between plant growth variables and seedling quality indices with physiological traits, soil microbial activity, soil enzymatic activities, and Si concentrations in plant tissues and soil. Correlations were calculated using R software (version 2024.12.1) with the corrplot package, and only significant correlations (*p* < 0.05) were presented.

## Results

### Growth parameters

**Fig. 1:**
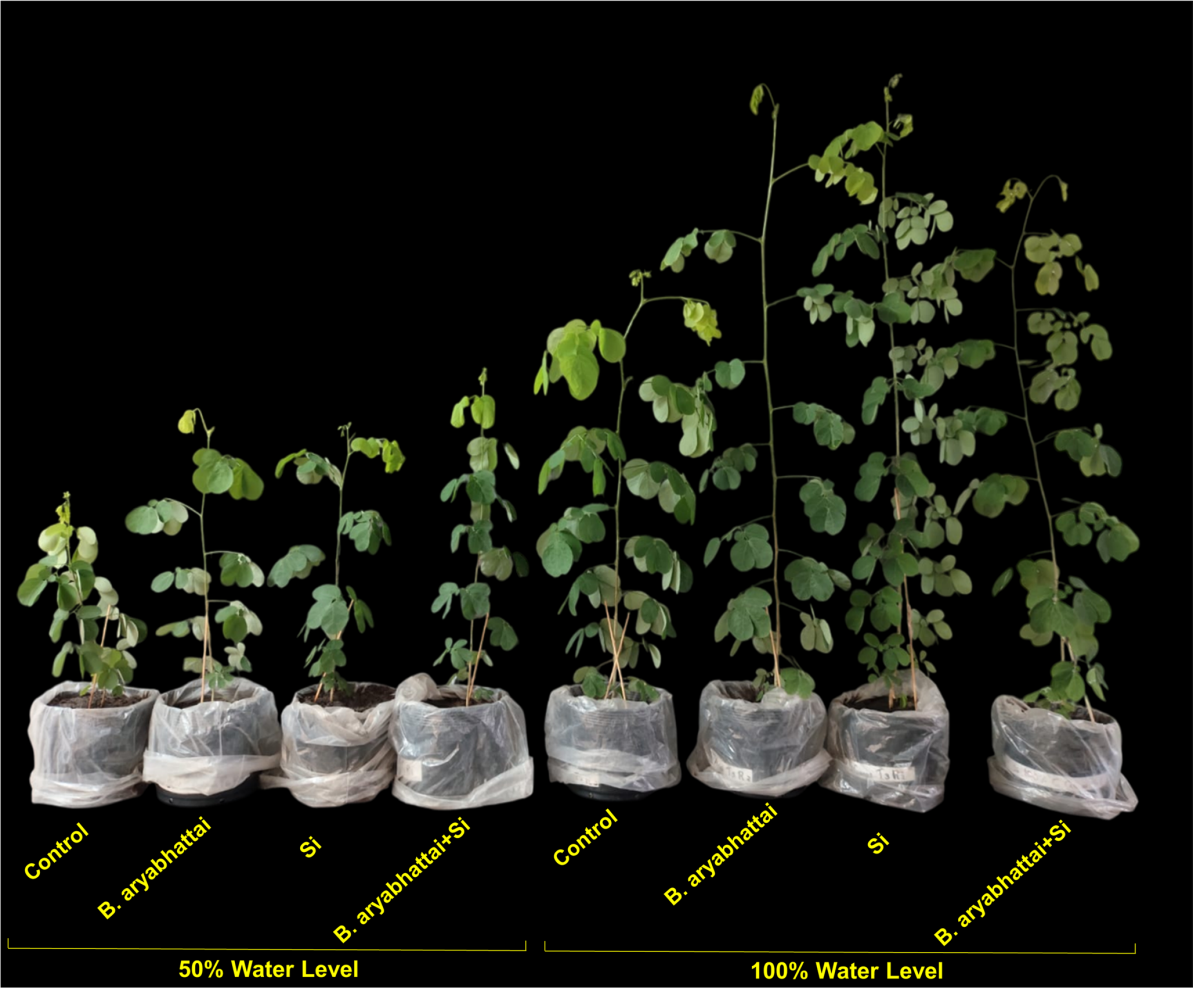
Growth of *M. caesalpiniifolia* seedlings under different water levels and treatments

**Fig. 2:**
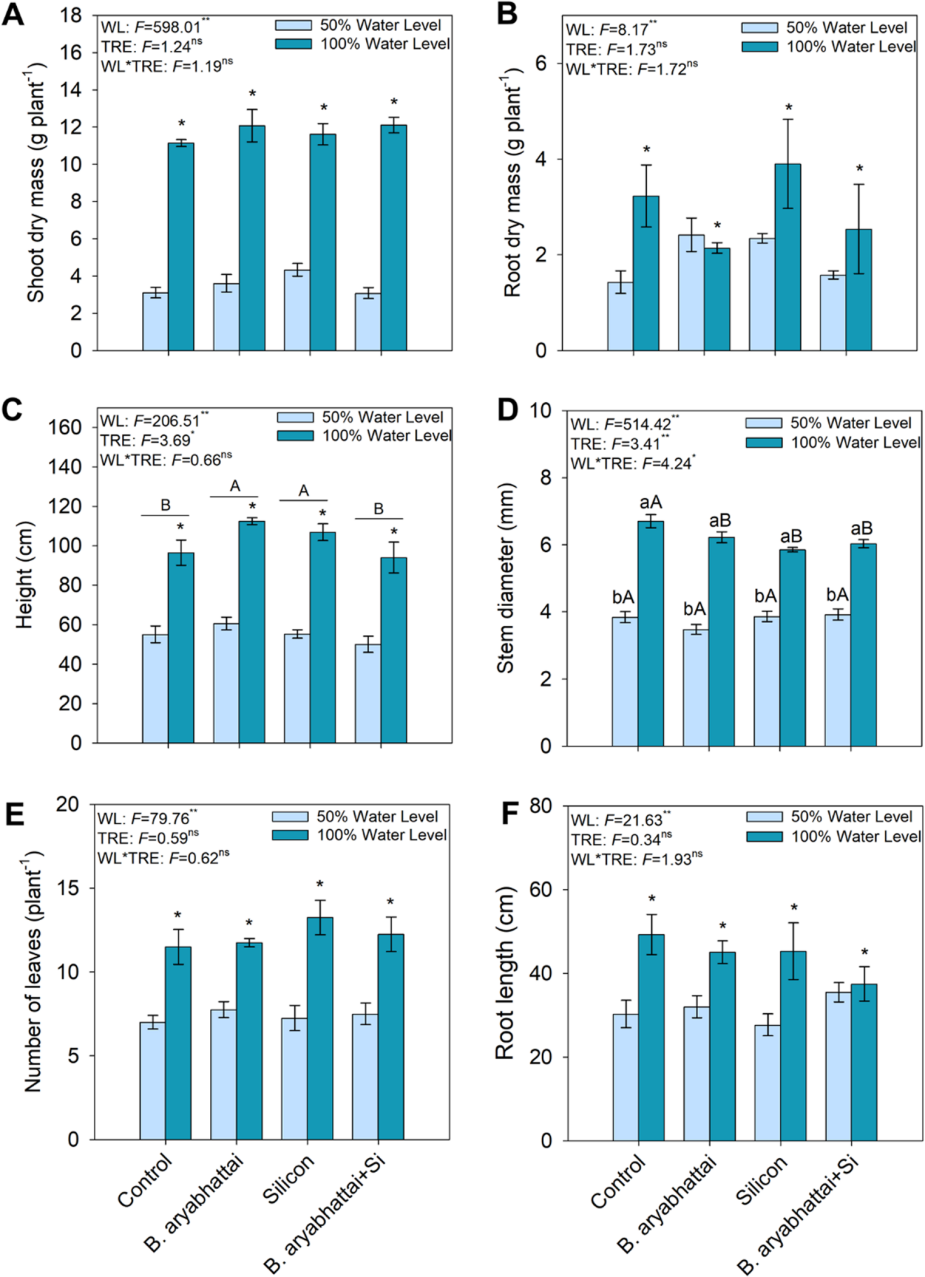
Growth parameters of *M. caesalpiniifolia* seedlings subjected to different treatments and water levels (50% and 100%). Shoot dry mass (**A**), root dry mass (**B**), height (**C**), stem diameter (**D**), number of leaves (**E**), and root length (**F**). Uppercase letters denote significant differences among treatments within the same water level (50% or 100%). When underlined, uppercase letters indicate significant differences among treatments regardless of water level. Lowercase letters and asterisks represent significant differences between plants grown under 50% and 100% water levels within the same treatment, based on the Scott-Knott test at a 5% significance level (*p* < 0.05)

The growth of *M. caesalpiniifolia* seedlings varied significantly according to the water levels and treatments (Fig. 1). Shoot and root dry mass, leaf number, and root length were greater in all treatments under 100% water level (Fig. 2A, B, E, F). Plant height was significantly higher in treatments with *B. aryabhattai* and Si applied individually under the 100% water level, with increases of ~ 13% and 7%, respectively, compared to the control (Fig. 2C). In contrast, stem diameter was consistently lower in all treatments under the 50% water level (Fig. 2D).

### Seedling quality

**Fig. 3:**
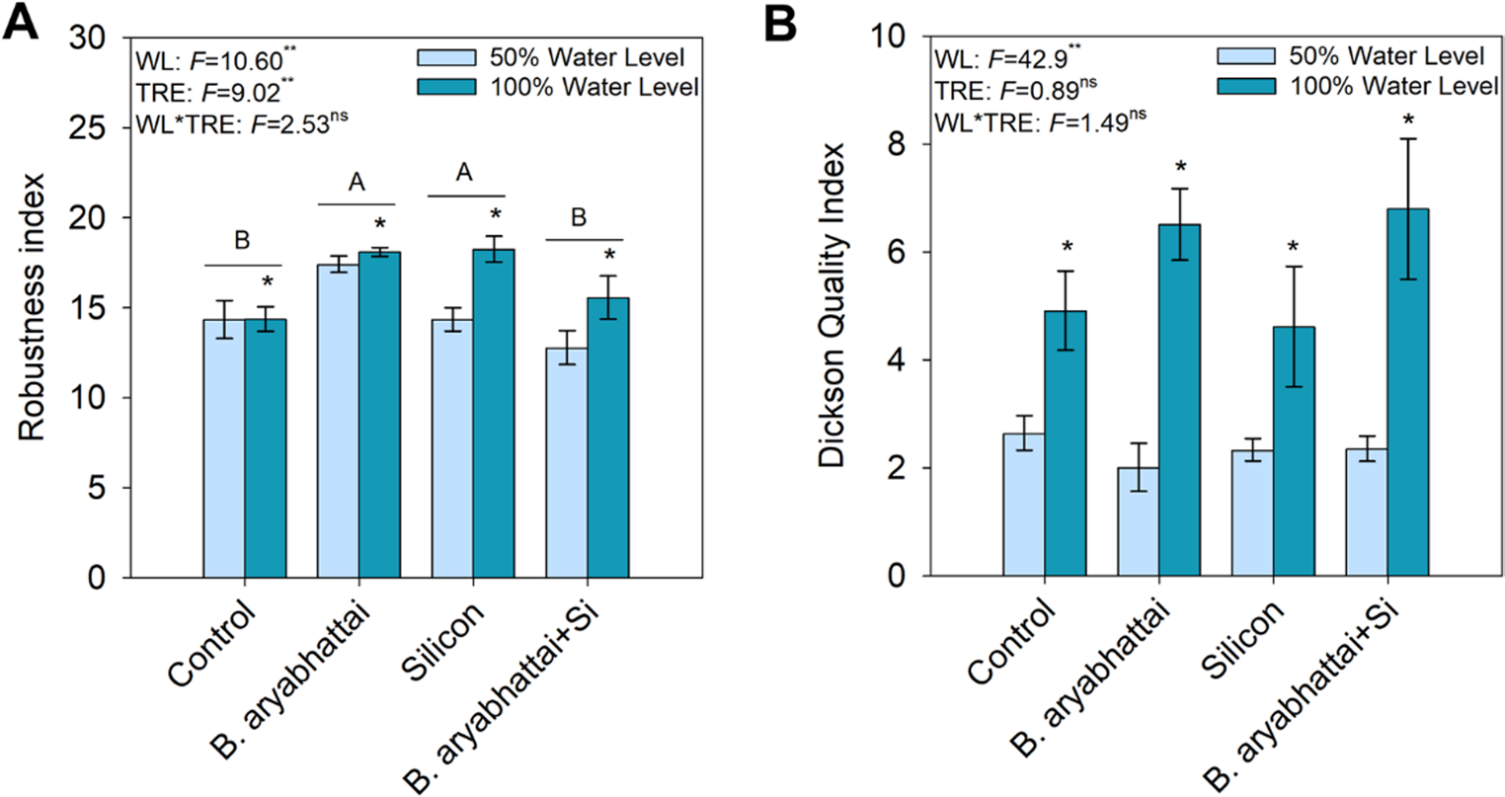
Robustness index (**A**) and Dickson quality index (**B**) in *M. caesalpiniifolia* subjected to different treatments and water levels. Underlined uppercase letters signify significant differences among treatments regardless of water level. Lowercase letters and asterisks denote significant differences between plants grown under 50% and 100% water levels within the same treatment, according to the Scott-Knott test at a 5% significance level (*p* < 0.05)

For the robustness index, seedlings subjected to 100% water level showed higher values across all treatments, with emphasis on the treatments with *B. aryabhattai* and Si applied individually (Fig. 3A). Similarly, the Dickson quality index was also higher under 100% water level in all treatments (Fig. 3B).

### Physiological parameters

**Fig. 4:**
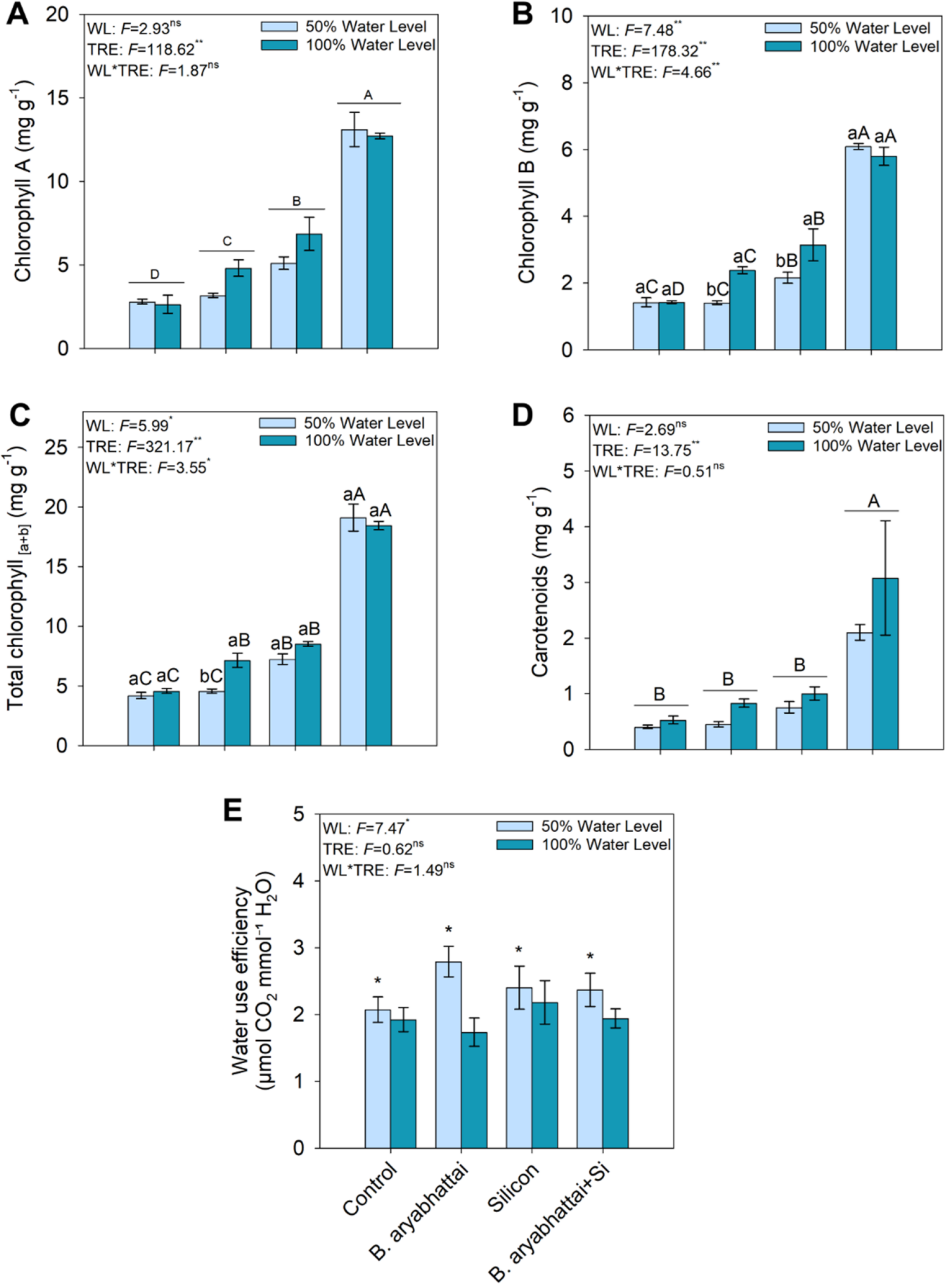
Physiological parameters in *M. caesalpiniifolia* subjected to different treatments and water levels: chlorophyll A (**A**), chlorophyll B (**B**), total chlorophyll (**C**), carotenoids (**D**), and water use efficiency (**E**). Uppercase letters denote significant differences among treatments within the same water level (50% or 100%). When underlined, uppercase letters indicate significant differences among treatments regardless of water level. Lowercase letters and asterisks represent significant differences between plants grown under 50% and 100% water levels within the same treatment, based on the Scott-Knott test at a 5% significance level (*p* < 0.05)

The highest levels of chlorophyll A, chlorophyll B, total chlorophyll, and carotenoids were observed in treatments with *B. aryabhattai* + Si, regardless of the water level (Fig. 4A, B, C, and D). On the other hand, water use efficiency (WUE) was higher under the 50% water level, regardless of Si or *B. aryabhattai* application (Fig. 4E).

### Microbial activity

**Fig. 5:**
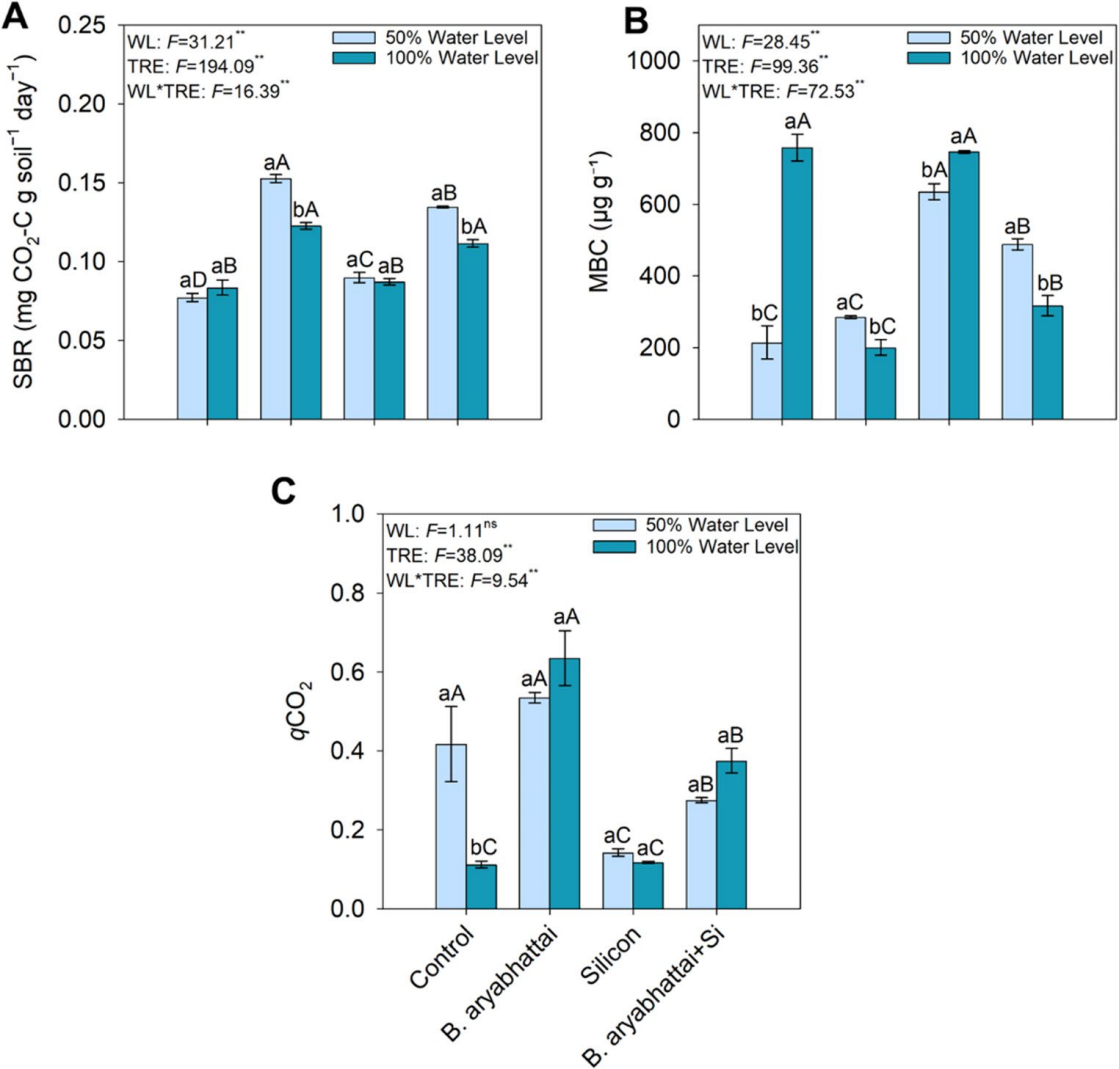
Soil basal respiration (SBR) (**A**), microbial biomass carbon (MBC) (**B**), and metabolic quotient (*q*CO₂) in soil subjected to different treatments and water levels. Uppercase letters indicate significant differences among treatments within the same water level (50% or 100%). Lowercase letters denote significant differences between plants grown under 50% and 100% water levels within the same treatment, based on the Scott-Knott test at a 5% significance level (*p* < 0.05)

Inoculation with *B. aryabhattai*, followed by the combined treatment with *B. aryabhattai* + Si, under the 50% water level, showed the highest mean values of SBR, with increases of ~ 87% and 75%, respectively, compared to the control (Fig. 5A). The highest values of MBC were observed in the control and Si treatments under the 50% water level (Fig. 5B), whereas the lowest *q*CO₂ was also recorded under these same conditions (Fig. 5C).

### Soil enzyme activity

**Fig. 6:**
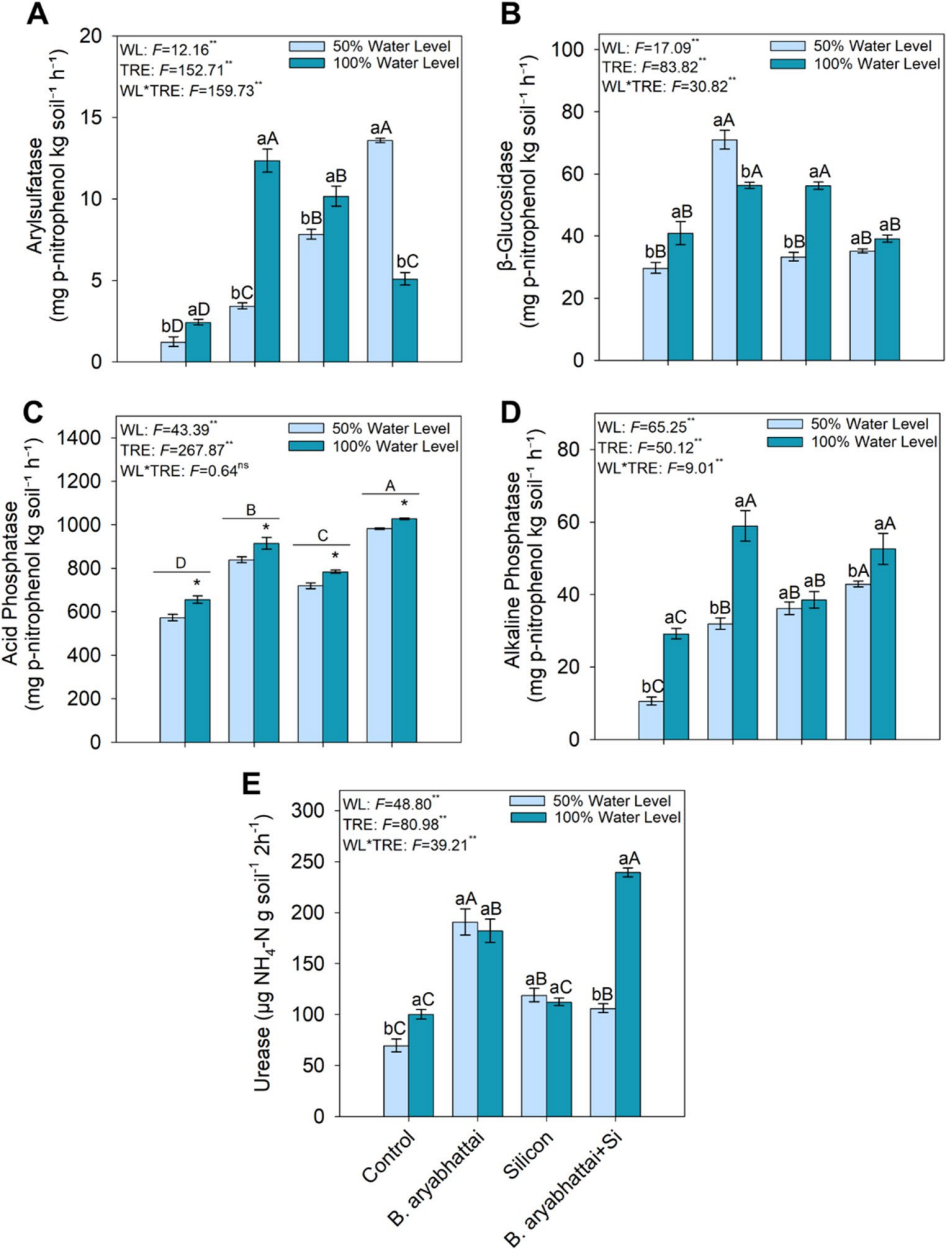
Activity of the enzymes Arylsulfatase (**A**), β-Glucosidase (**B**), Acid Phosphatase (**C**), Alkaline Phosphatase (**D**), and Urease (**E**) in soil subjected to different treatments and water levels. Uppercase letters denote significant differences among treatments within the same water level (50% or 100%). When underlined, uppercase letters indicate significant differences among treatments regardless of water level. Lowercase letters and asterisks represent significant differences between plants grown under 50% and 100% water levels within the same treatment, based on the Scott-Knott test at a 5% significance level (*p* < 0.05)

Soil arylsulfatase activity was highest in the treatment with *B. aryabhattai* + Si under the 50% water level (Fig. 6A), while β-glucosidase activity was greatest in the treatment with *B. aryabhattai* alone under the same condition (Fig. 6B), with increases of ~ 600% and 140%, respectively, compared to the control. Acid phosphatase activity was higher in the treatment with *B. aryabhattai* + Si regardless of water level, and also under the 100% water level regardless of *B. aryabhattai* or Si application (Fig. 6C). The presence of *B. aryabhattai* in the treatments corresponded to higher alkaline phosphatase activity, alone and in combination with Si under the 100% water level, showing increases of ~ 75% and 88%, respectively, in relation to the control (Fig. 6D). Urease activity was highest in the treatment with *B. aryabhattai* + Si under the 100% water level, reflecting an ~ 100% increment in relation to the control (Fig. 6E).

### Silicon content in shoots, roots, and soil

**Fig. 7:**
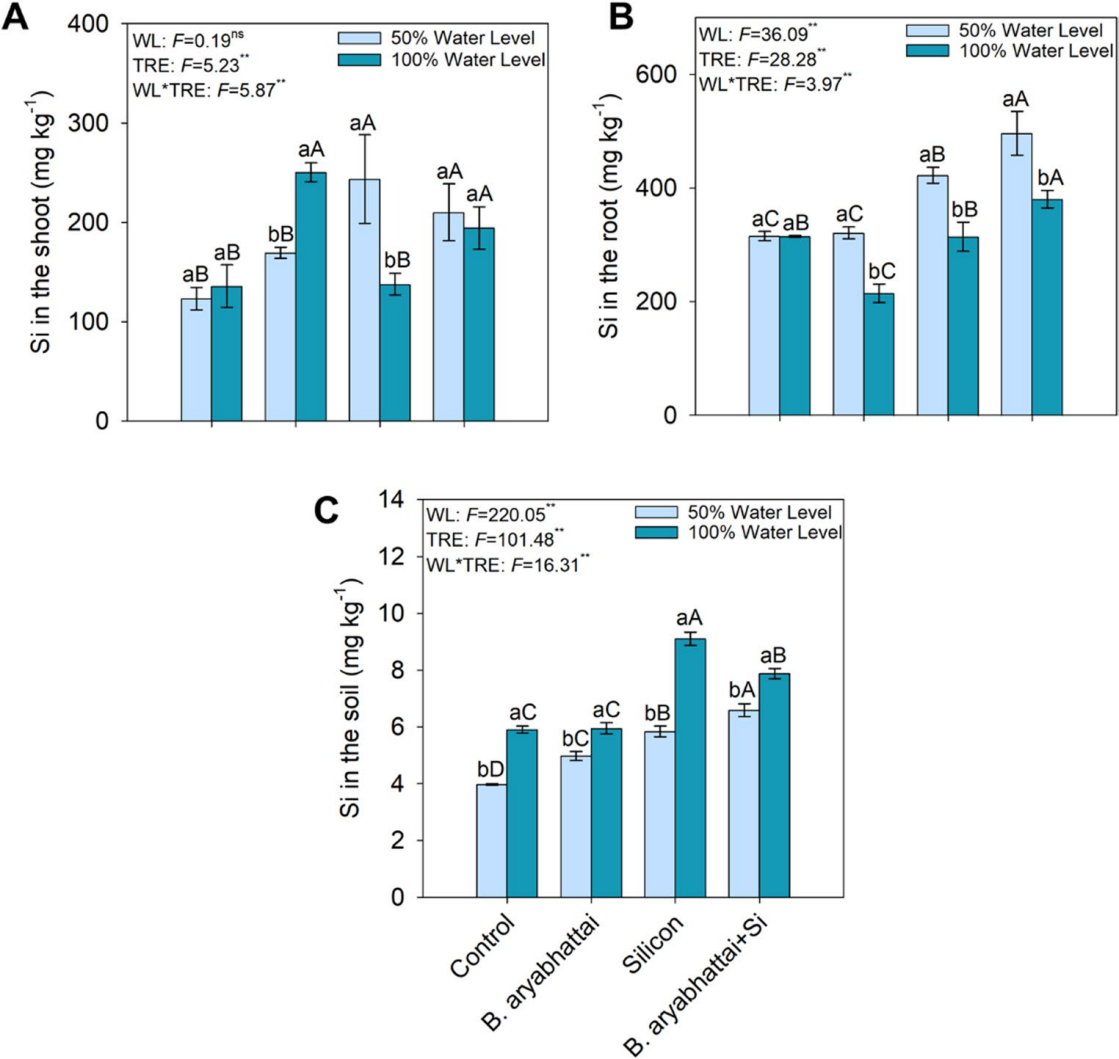
Silicon content in the shoot (**A**) and root (**B**) of *M. caesalpiniifolia*, and in the soil (**C**), under different treatments and water levels. Uppercase letters indicate significant differences among treatments within the same water level (50% or 100%). Lowercase letters denote significant differences between plants grown under 50% and 100% water levels within the same treatment, based on the Scott-Knott test at a 5% significance level (*p* < 0.05)

Si concentration in the shoot was highest in the treatment with *B. aryabhattai* under the 100% water level (Fig. 7A). Within the roots, the highest Si concentration occurred in the treatment combining *B. aryabhattai* + Si under the 50% water level, followed by the treatment with Si alone under the same condition (Fig. 7B). Within the soil, the highest Si concentration was observed in the treatment with Si alone under the 100% water level (Fig. 7C).

### Physiological, soil biological, and silicon relationships with plant growth under contrasting water levels

**Fig. 8:**
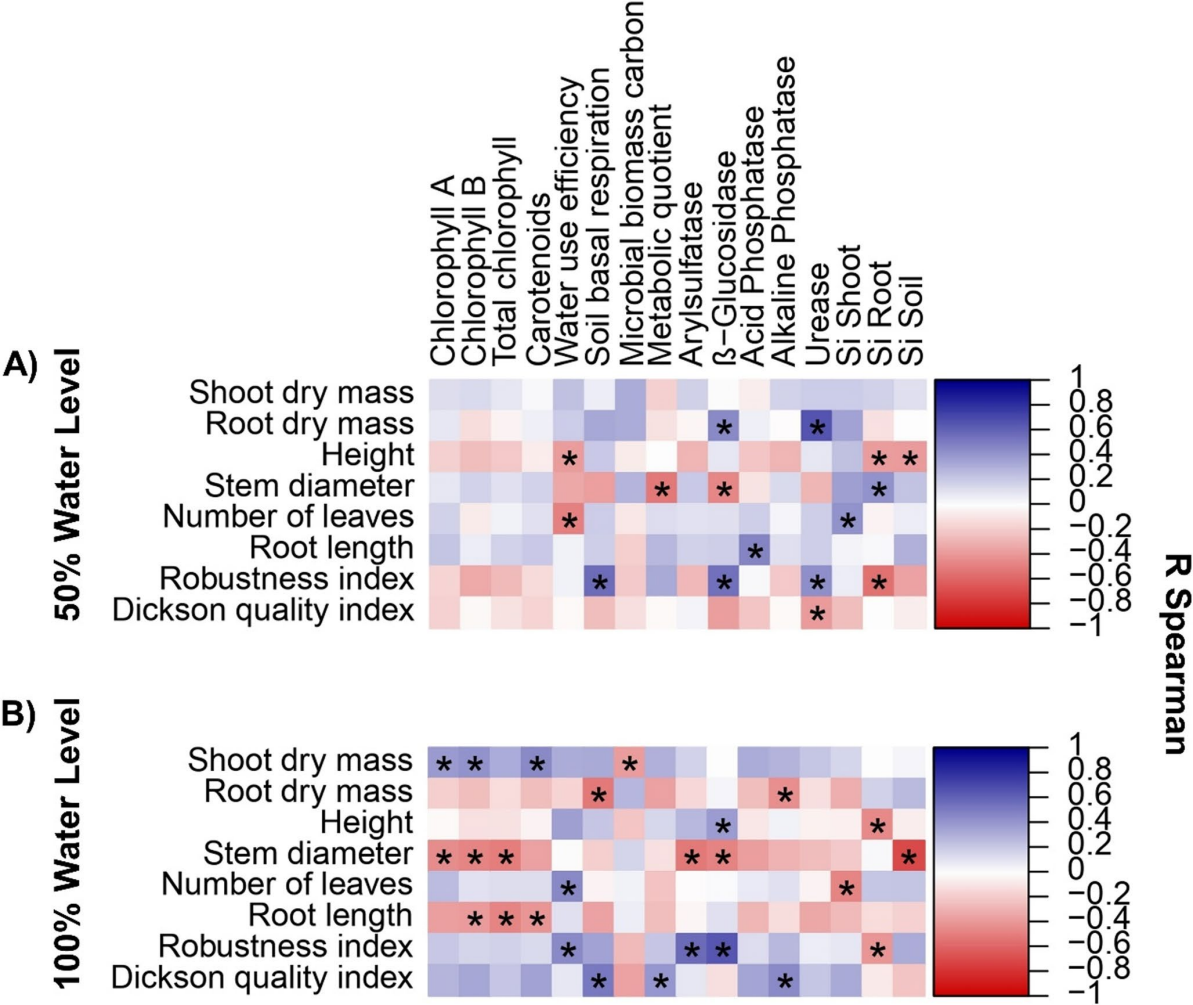
Spearman correlation heatmaps showing relationships between plant growth and seedling quality traits and physiological parameters, soil microbial activity, soil enzymatic activities, and silicon concentrations in plant tissues and soil under different water regimes: (**A**) 50% water level and (**B**) 100% water level. Color intensity represents the strength and direction of correlations (blue = positive, red = negative). Asterisks indicate significant correlations (*p* < 0.05). Si shoot, Si root, and Si soil = silicon concentrations in shoot, root, and soil, respectively

Spearman correlations showed associations among plant growth, physiological traits, soil enzymatic activities, microbial attributes, and silicon fractions under contrasting water levels (Fig. 8). At 50% water level (Fig. 8A), significant correlations were scarce. Plant growth traits had limited associations with soil biological attributes and were mostly negative for height, stem diameter, and number of leaves. Root dry mass showed isolated positive correlations with β-glucosidase and urease. The robustness index was positively correlated with soil basal respiration, β-glucosidase, and urease, whereas the Dickson Quality Index showed a single negative correlation with urease. Positive associations were also observed between root Si and stem diameter and between shoot Si and number of leaves. At 100% water level (Fig. 8B), significant correlations were more frequent. Shoot dry mass was positively correlated with photosynthetic pigments. In contrast, stem diameter showed negative correlations with β-glucosidase and arylsulfatase, while root length was negatively correlated with photosynthetic pigments. The robustness index was positively correlated with arylsulfatase, β-glucosidase, and water use efficiency, and the Dickson Quality Index was positively correlated with soil basal respiration and alkaline phosphatase. Negative associations between silicon fractions and plant growth traits were more frequent than at 50% water level.

## Discussion

In this study, we assessed the potential of applying *B. aryabhattai* and Si to promote the growth and physiological performance of *M. caesalpiniifolia* seedlings, while also influencing soil biological activity, under different levels of water stress. Our main findings confirmed that water availability is crucial for the development of *M. caesalpiniifolia*, markedly impacting its growth parameters. Indeed, we observed a significant reduction in shoot and root dry mass, as well as in leaf number and root length under water stress conditions. Although the application of *B. aryabhattai* and Si, separately and combined, showed relatively positive effects; however, water limitation remained the dominant factor, suggesting that early-stage vegetative growth of *M. caesalpiniifolia* is particularly sensitive to water scarcity. This sensitivity may be related to the physiology of seedlings, which is potentially geared toward rapid initial growth [40].

Although Si is widely recognized for its role in mitigating abiotic stresses, its isolated or combined application with *B. aryabhattai* did not significantly improve the evaluated morphological traits. This response is likely associated with constraints on Si transport within the plant under low transpiration conditions [41]. The high Si concentration observed in roots, especially under 50% water level, supports this hypothesis, indicating preferential allocation to the root system. Correlation analysis further reinforced this interpretation, revealing that root Si was more strongly associated with growth traits under water deficit, suggesting restricted Si translocation to the shoot. This limited distribution may restrict Si movement to the shoot, where key processes related to photosynthesis and the regulation of water loss occur [42]. Furthermore, it is important to consider that plants inoculated with rhizobacteria typically show more pronounced physiological and morphological responses after prolonged cultivation periods, often exceeding 90 days [43]. Reported that the most expressive effects of such symbiotic interactions tend to occur between 120 and 270 days after inoculation, which may partly explain the absence of more significant differences among treatments in this early phase study.

The robustness index and Dickson quality index are widely recognized as essential indicators of vigor and quality in forest seedlings [25, 26]. According to the criteria established by [44], Dickson quality index values lower than 0.2 suggest that the seedlings are not appropriate for transplantation in the field, whereas higher values are associated with greater potential for survival and establishment. In the present study, the Dickson index showed higher values under 100% water level conditions, regardless of Si application or *B. aryabhattai* inoculation. Although the application of Si or *B. aryabhattai* has been shown to promote tolerance to abiotic stresses [5, 44], their isolated or combined effects were not sufficient to mitigate the impact of water limitation on seedling quality traits. On the other hand, the robustness index varied significantly in response to treatments with *B. aryabhattai* and Si applied individually. This variation is likely linked to increases in seedling height, a parameter directly included in the index calculation. Nevertheless, the observed gains were not sufficient to overcome the constraints imposed by water deficit. These findings reinforce that water availability was the main determinant of *M. caesalpiniifolia* seedling quality, with Si and inoculant contributions being marginal under severe stress conditions.

From a physiological perspective, plant responses were among the most sensitive to treatment interactions. Water use efficiency was higher under water stress in our study, which can be attributed to stronger stomatal restriction, a common adaptive response in plants to reduce water loss through transpiration [45]. While this mechanism aids in water conservation, it also limits CO_2_ uptake, compromising the balance between C fixation and water loss. Thus, increased water use efficiency does not necessarily translate into higher biomass under severe drought. In addition, in our study, we found higher concentrations of chlorophylls and carotenoids in plants subjected to the combined application of Si and *B. aryabhattai*, regardless of water availability. Positive correlations between pigments and shoot biomass, particularly under adequate water supply, indicate that pigment preservation contributed to effective biomass accumulation. This pattern suggests a synergistic effect in preserving pigments related to photosynthesis, possibly linked to the attenuation of oxidative damage or the activation of antioxidant mechanisms [46]. Similar results were described by [47], who reported higher relative water content and lower pigment degradation in *Zea mays* under water deficit after Si application or *B. aryabhattai* inoculation. Likewise [48], observed increased chlorophyll and carotenoid levels in legume plants treated with Si under moderate stress, suggesting a protective effect mediated by membrane stability and reduced lipid peroxidation.

Soil microbial activity indicators revealed functional interactions between Si and *B. aryabhattai* in C dynamics. Correlations further indicated that microbial biomass and enzyme activities were positively associated with shoot biomass and seedling quality indices under 100% water level, suggesting a tighter association between plant and microbial variables when water was not limiting. Increased SBR in treatments with *B. aryabhattai*, alone or combined with Si under 50% water level, suggests enhanced microbial metabolic activity even under water limitation. This response may be associated with increased root exudation induced by *B. aryabhattai*, which can supply readily assimilable substrates to the microbiota, in addition to the possible microbial production of exopolysaccharides, which enhance water retention in the rhizosphere and protect microbial cells [43, 49]. Conversely, higher MBC in control and Si-only treatments under drought suggests a conservative microbial strategy, with greater carbon stabilization and lower turnover [49]. This interpretation aligns with the low *q*CO₂ values observed, indicating higher microbial carbon-use efficiency [50]. These results suggest that even when foliar Si uptake is limited under water deficit, the element can exert beneficial indirect effects on the soil ecosystem. Thus, the interaction between Si and *B. aryabhattai* tends to create more stable and protected microenvironments, promoting greater metabolic efficiency and microbial resilience under water stress.

Soil enzyme activity was significantly modulated by the interaction between *B. aryabhattai* inoculation and Si application, particularly under 50% water level. Arylsulfatase, an enzyme involved in the mineralization of organic sulfur compounds, showed a significant increase, suggesting greater release of available sulfur. This effect may be linked to rhizosphere microbial community modulation in response to synergistic stimuli from the consortium [51]. β-glucosidase activity was significantly stimulated in the *B. aryabhattai* only treatment, indicating increased saprophytic activity and more intense C dynamics in the soil. Acid phosphatase, responsible for releasing organic P, showed maximum activity in the combined treatment with *B. aryabhattai* and Si, demonstrating that this association promotes P access regardless of water regime [52]. Similarly, alkaline phosphatase and urease showed higher activity under the joint application of these agents, highlighting that enzyme response is specific to each enzyme and target nutrient and is also modulated by soil water availability. These results reinforce the potential of the consortium between Si and plant-growth-promoting microorganisms as a functional catalyst in the rhizosphere, enhancing the availability of essential nutrients even under water restriction [53]. Therefore, the combined application of these agents in water-limited soils may contribute to soil conditioning, stimulating key biochemical processes for nutrient cycling and improving biological fertility. Consequently, this management approach may support the recovery of soil quality and revegetation of degraded areas, creating more favorable conditions for the establishment and growth of other plant species in adverse environments.

Si concentrations in the plant and soil revealed differential allocation as a function of water level and treatment type. Higher Si concentration in shoot was observed only under 100% water level, especially in the *B. aryabhattai* treatment, suggesting that element absorption and transport to foliar tissues depend directly on transpiration flow and root activity associated with rhizospheric microbiota [54]. Under water stress, water mass flow is restricted, limiting Si transport through the xylem and resulting in its retention in the roots [55]. This retention may provide structural protection to roots, preventing cell collapse, promoting the development of thicker exodermis layers, and modulating root exudation. On the other hand, higher silicon availability in the soil observed in the Si treated plots under 100% water level indicates that part of the element remained in the soil solution but was not necessarily absorbed in greater quantity, reinforcing the role of the water gradient in active Si transport [56]. The physiological effects of Si are strongly dependent on water regime, being more effective under conditions that favor transpiration, the main driver of its transport, and less effective under severe water stress [57]. This behavior was also described [58], who observed higher Si accumulation in the roots of *Triticum aestivum* under water deficiency, with low translocation to shoot due to reduced transpiration flow. Similarly [59], reported that under stress conditions, Si tends to accumulate in root tissues, promoting cell wall thickening and protecting cells from desiccation, while its translocation to the shoot remains limited. Overall, these findings demonstrate that Si effectiveness is strongly modulated by plant water status, with greater physiological expression under conditions that sustain transpiration flow.

## Conclusions

The growth and quality of *M. caesalpiniifolia* seedlings were significantly affected by water restriction, highlighting the species’ sensitivity to drought stress. Seedlings grown under water restriction showed marked reductions in biomass accumulation and quality indices, reinforcing the importance of adequate water supply for successful establishment. The joint use of *B. aryabhattai* and Si was associated with greater physiological performance and shifts in soil enzymatic and microbial functioning, with the magnitude of change depending on both the enzyme assessed and soil moisture conditions. The findings indicate that the combination of *B. aryabhattai* and Si can enhance soil biological functioning and promote more favorable rhizosphere conditions, even when plant growth gains are limited by drought intensity. This interaction shows potential to produce more resilient seedlings in semiarid environments by promoting soil functional recovery, a key factor for ecological restoration. The effectiveness of this approach depends on drought intensity, and further studies across species and longer cultivation periods are needed to determine its consistency and field applicability under semiarid conditions.

## Data Availability

Raw data were generated in the Soil Microbiology Laboratory of the Soil Science Department of the Federal University of Ceará, Fortaleza, Brazil. Derived data supporting the findings of this study are available from the corresponding author [K.G.V. Garcia] upon request.
